# Entwicklung zweier ambulanter gruppentherapeutischer interdisziplinärer Therapiemodule in der Behandlung von Patienten mit Schmerzen und Chronifizierungsrisiko

**DOI:** 10.1007/s00482-023-00692-y

**Published:** 2023-02-23

**Authors:** Anke Preißler, Leonie Schouten, Greta Hoffmann, Karin Deppe, Gabriele Lindena, Frank Petzke, Anne Gärtner, Ulrike Kaiser

**Affiliations:** 1 UniversitätsSchmerzCentrum, Klinik für Anästhesiologie, Universitätsklinikum Carl Gustav Carus Dresden, Fetscherstraße 74, 01307 Dresden, Deutschland; 2 Schmerzmedizin, Klinik für Anästhesiologie, Universitätsmedizin Göttingen, Robert-Koch-Straße 40, 37075 Göttingen, Deutschland; 3 Klinik für Anästhesiologie und Intensivmedizin, Universitätsklinikum Schleswig-Holstein, Ratzeburger Allee 160, 23538 Lübeck, Deutschland; 4 Deutsche Schmerzgesellschaft e. V., Berlin, Deutschland

**Keywords:** Sekundärprävention, Schmerzmanagement, Therapiemanual, Interdisziplinäre multimodale Therapie, Wirkmodell, Secondary prevention, Pain management, Treatment manual, Interdisciplinary pain management, Causal model

## Abstract

**Hintergrund und Zielsetzung:**

Eine frühe Versorgung von Patient:innen, die Schmerzen und Risikofaktoren einer Chronifizierung aufweisen, ist sinnvoll; diese Patient:innen können von einer frühen interdisziplinären multimodalen Schmerztherapie (IMST) profitieren. Angesichts der unzureichenden Versorgung wurden im Rahmen von PAIN2020 zwei ambulante Therapiemodule für die Sekundärprävention einer Schmerzchronifizierung entwickelt werden: die edukative bzw. begleitende IMST (E‑IMST/B‑IMST).

**Material und Methoden:**

Der Entwicklungsprozess der beiden IMST wird vorgestellt. Es wurden zwei Zielgruppen von Patient:innen definiert, für die in Abhängigkeit von Chronifizierung, Beeinträchtigung und Komplexität der Störung eine Sitzung (E‑IMST) bzw. 10 Interventionstermine (B‑IMST) vorgesehen waren. Die Konzeption erfolgte in 5 Schritten: Erarbeitung der Zielstellungen; Erarbeitung der Hauptinhalte; Workshop zur inhaltlichen und konzeptionellen Ausgestaltung (Inhalte, Vermittlung, Übungen); Erstellung eines Ablaufplans; Aufbereitung der Ergebnisse (Manual, Präsentationen, Arbeitsblätter, Handbuch). Zunächst wurde die B‑IMST entwickelt, woraus Inhalte für die E‑IMST extrahiert wurden. Daneben sollte ein Konzept zur Überprüfung der Umsetzbarkeit und ein Wirkmodell für eine Pilotierung entwickelt werden.

**Ergebnisse:**

Zielstellungen für beide IMST-Formen sind die Verbesserung des Verständnisses von Schmerz und mitbedingenden Faktoren, die Erhöhung des Kontroll- und Selbstwirksamkeitserlebens und die Erhöhung der Eigenverantwortung hinsichtlich schmerzreduzierender Strategien. Unterschiede zwischen den beiden Therapiemodulen ergeben sich aus den Bedarfen und Rahmenbedingungen. Für beide IMST-Module wurden ärztliche, physio- und psychotherapeutische Inhalte und Abläufe ausgestaltet. Die B‑IMST setzt sich aus 5 Modulen mit je 2 Terminen als Gruppenintervention zusammen (biopsychosoziales Modell; Aktivierungsplanung; Bedürfnisregulation; Schlaf und Medikamente; Alltagstransfer). Die 3‑stündige E‑IMST-Gruppenintervention vermittelt in erster Linie Wissen über Schmerz und das biopsychosoziale Schmerzmodell. Es kommen theoretische und praktische Interventionen bzw. erfahrungs- und erlebnisorientierte Methoden zur Anwendung.

**Schlussfolgerung:**

Es existieren nun zwei interdisziplinär ausgerichtete Manuale für die sekundärpräventive Behandlung von Patient:innen mit wiederkehrenden Schmerzen und Risikoprofil für eine Chronifizierung. Diese Ansätze müssen nun hinsichtlich Machbarkeit und Wirksamkeit überprüft werden.

## Abstract (en)

**Background and objective:**

An early treatment of patients who suffer from pain and show risk factors for chronification is meaningful as these patients can benefit from an early interdisciplinary multimodal pain treatment (IMST). In view of the insufficient treatment, two outpatient treatment modules for the secondary prevention of pain chronification are developed within the framework of PAIN2020: the educative and accompanying IMST (E‑IMST and B‑IMST).

**Material and methods:**

The developmental process of both IMSTs is presented. In this context two target groups of patients were defined for which 1 session (E‑IMST) or 10 intervention appointments (B‑IMST) were planned, depending on the chronification, impairment and complexity of the disorder. The conception was carried out in five steps: development of the objectives, development of the main contents, workshop on the content and conceptional design (contents, mediation, exercises), preparation of a time schedule and processing of the results (manual, presentations, worksheets, handbook). The B‑IMST was initially developed from which the contents for the E‑IMST were subsequently extracted. Additionally, a concept for testing the feasibility and a working model for a pilot study were developed.

**Results:**

The objectives for both forms of IMST are improvement of the understanding of pain and contributing factors, the increase of the experience of control and self-efficacy and the increase in self-responsibility with respect to strategies to reduce pain. Differences between the two treatment modules arise from the needs and framework conditions. Medical, physiotherapeutic and psychotherapeutic contents and schedules were organized for both IMST modules. The B‑IMST consists of five modules each with two sessions as group interventions (biopsychosocial model, activation planning, regulation of needs, sleep and medication, routine transfer). The 3‑h E‑IMST group intervention mainly intends to mediate knowledge on pain and the biopsychosocial pain model. Theoretical and practical interventions, empirical knowledge and experience-oriented methods are employed.

**Conclusion:**

There are now two interdisciplinary structured manuals for the secondary preventive treatment of patients with recurrent pain and a risk profile for chronification. These approaches must now prove themselves with respect to feasibility and effectiveness.

Die Prävention chronischer Schmerzverläufe ist in den letzten Jahren stärker in den Fokus gerückt, wie sich beispielsweise im Global Year for the Prevention of Pain (GY) der International Association for the Study of Pain (IASP; [12]), in der Sonderausgabe von *Der Schmerz* zum GY der IASP im Jahr 2021 oder in abgeschlossenen bzw. laufenden Projekten zeigt (PAIN2020/01NVF17049, PAIN2.0/01NVF20023, Poet-Pain/01NVF19021).

Frühe Versorgung von Patient:innen mit Schmerz und Risikofaktoren einer Chronifizierung ist sinnvoll

Von wissenschaftlicher Seite wird zunehmend die frühzeitige Behandlung von Patient:innen mit wiederkehrenden Schmerzen durch interdisziplinäre Ansätze in Diagnostik und Therapie gefordert, die bekannte Risiko- und psychosoziale Begleitfaktoren berücksichtigen [3]. Dies bezieht sich zunächst auf Patient:innen mit Rückenschmerzen, bei denen eine Reduktion ihrer Schmerzen und der schmerzbedingten Beeinträchtigung nicht nur den Schweregrad und die persönlichen Auswirkungen auf die Lebensqualität, sondern auch langfristig die Aufwendungen der Kostenträger und des Gesundheitswesens reduzieren konnte [26, 36, 38].

Aus den Erfahrungen schmerzmedizinischer Einrichtungen mit Patient:innen mit unterschiedlichen Schmerzlokalisationen [1, 29, 31, 34] lässt sich ableiten, dass eine frühe Versorgung von Patient:innen, die Schmerzen und Risikofaktoren einer Chronifizierung aufweisen, sinnvoll ist und dass diese ebenso wie Patient:innen mit chronischen Schmerzen von einer frühen interdisziplinären multimodalen Schmerztherapie (IMST) profitieren könnten. Im Rahmen eines Innovationsfondsprojekts für neue Versorgungsformen (NVF) wurden daher zwei Therapiemodule entwickelt.

## Primär‑, Sekundär- und Tertiärprävention

**Figure 1:**
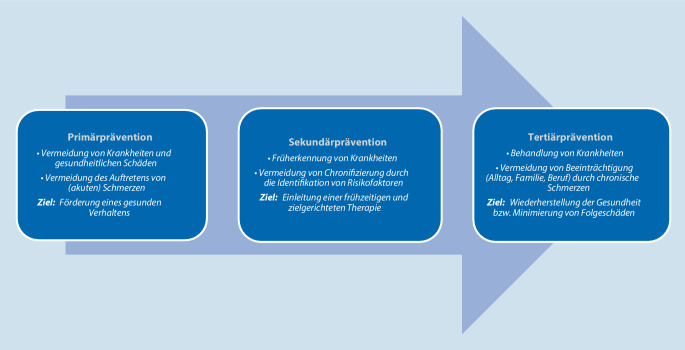

Um die zeitlichen Ansatzpunkte in der Diagnostik und Therapie von Schmerzen zu unterscheiden, werden im internationalen Sprachraum die Begrifflichkeiten der Primär‑, Sekundär- und Tertiärprävention verwendet (vgl. Abb. 1). Während es bei der Primärprävention um die Vermeidung eines Auftretens akuter Schmerzen geht, versucht die Sekundärprävention, wiederkehrende Schmerzen so zu erkennen und zu behandeln, dass sich daraus kein chronifizierendes Geschehen entwickelt. Die Tertiärprävention bezieht sich demgegenüber nach internationalem Verständnis auf das Vermeiden einer fortschreitenden Beeinträchtigung durch eingetretene chronische Schmerzverläufe. Dies entspricht im deutschen Gesundheitswesen einer Behandlung und würde hier nicht als Prävention verstanden werden [19, 20]. In diesem Beitrag werden zwei Interventionen im Rahmen der Sekundärprävention vorgestellt.

## Aktueller Stand der Sekundärprävention in Deutschland

Im Health-Technology-Assessment-Bericht [8] wird die derzeitige Versorgungssituation von Patient:innen mit Schmerzen als unzureichend beschrieben und durch eine *Über*versorgung (u. a. unimodale und medizinische Diagnostik), *Fehl*versorgung (u. a. fehlende Steuerung in bedarfsgerechte Therapieangebote und mangelnde Anwendung bestehender Leitlinien) und *Unter*versorgung (u. a. im Hinblick auf aktivierende, psychotherapeutische oder interdisziplinäre Angebote zur Vermeidung des Chronifizierungsprozesses einer Schmerzerkrankung) charakterisiert. Vor allem im ambulanten Sektor der Regelversorgung werden Menschen mit (häufig) wiederkehrenden Schmerzen und Risikofaktoren für eine Chronifizierung nach wie vor eher unimodal in der hausärztlichen oder fachärztlichen Versorgung, z. B. in der Orthopädie oder Neurologie, betreut [3, 4, 21]. Ergänzend ist weder eine integrierte Diagnostik (interdisziplinäres multimodales Assessment [IMA]; [5]) noch eine berufs- und alltagsbegleitende, ambulant durchgeführte Form der IMST abgebildet [14, 15].

Vonseiten der Deutschen Schmerzgesellschaft e. V. existieren bereits zahlreiche Empfehlungen zur Umsetzung interdisziplinärer multimodaler Diagnostik- und Therapieansätze für Patient:innen mit chronischen Schmerzen, die sich möglicherweise auf Patient:innen mit Schmerzen und Chronifizierungsrisiko im Rahmen der Sekundärprävention übertragen ließen [1, 2, 5, 28].

Aktuelle Studien zur Prävention einer Schmerzchronifizierung (Sekundärprävention) gibt es wenige [9, 17]. In einzelnen Projekten (meist standortbezogen, nicht flächendeckend) zur Sekundärprävention von Schmerzen im ambulanten Bereich zeigt sich, dass bei Patient:innen mit wiederkehrenden Schmerzen durch bedarfsgerechte Versorgung die schmerzbezogenen Beschwerden und die damit verbundenen Kosten reduziert werden können [4, 18, 21] bzw. dass an Risikoprofilen orientierte Kurzinterventionen hinsichtlich schmerzbedingter Beeinträchtigung und Funktionsfähigkeit erfolgreich sind [6, 11]. Die Kernelemente der Projekte fokussierten sich dabei auf eine frühzeitige Identifikation von Versicherten mit Rückenschmerz, eine interprofessionelle Diagnostik und anschließende ambulante interprofessionelle Versorgungsangebote mit ausreichender Intensität, die an Risikofaktoren orientiert und unter Berücksichtigung des Chronifizierungsgrads entwickelt wurden.

Systematische Literaturübersichten zu Programmen mit Patient:innen, die Rücken- und muskuloskeletale Schmerzen aufweisen, zeigen, dass eine Kombination aus Edukation (Vermittlung von Wissen über die multikausale Genese von Schmerz) und angeleiteten körperlichen Übungen für kurz- und mittelfristige Zeiträume gute Effekte in der Reduktion von Schmerz und Funktionsbeeinträchtigung erzielen kann [22, 23, 33, 37].

## Zielsetzung

Da bisher ein an den Versorgungsbedarf angepasstes Therapieprogramm für Menschen mit wiederkehrenden Schmerzen und Risikoprofil für eine Chronifizierung fehlt (u. a. aufgrund fehlender evidenzbasierter Therapieprogramme und mangelnder finanzieller Vergütung), wurden im Rahmen von PAIN2020 – ergänzend zur Regelversorgung nach dem IMA [15] – zwei ambulante interdisziplinäre multimodale Therapiemodule konzipiert: die *edukative* (E‑IMST) und die *begleitende* interdisziplinäre multimodale Schmerztherapie (B‑IMST).

Mit der vorliegenden Arbeit soll der Entwicklungsprozess – insbesondere die Konzeption und die Erstellung des Therapiemanuals – beider ambulanter, die Regelversorgung ergänzender interdisziplinärer multimodaler Therapieangebote für Patien:innten mit Schmerzen und Chronifizierungsrisiko vorgestellt werden.

## Methoden – Entwicklungsprozess

### Empfehlungen zur Entwicklung komplexer Interventionen

Ausgehend von aktueller, wenn auch noch spärlicher Evidenz [22, 23, 33, 37] legt die Kombination von Edukation und angeleiteten körperlichen/praktischen Übungen zur Vermeidung chronischer Schmerzverläufe die Anwendung interdisziplinärer Versorgungsangebote nahe. Dabei fallen solche Angebote in den Bereich komplexer Interventionen, die beispielsweise durch die Menge an verschiedenen Interventionen, deren mögliche Interaktion bzw. durch die Menge an verschiedenen erforderlichen Kompetenzen der Behandelnden zur Durchführung dieser Intervention charakterisiert werden [7]. Darüber hinaus werden komplexe Interventionen auch als ein Ereignis in Systemen verstanden, die sowohl durch Kontextfaktoren beeinflusst werden als auch wiederum ihrerseits Kontexte beeinflussen [32].

Die Entwicklung komplexer Interventionen durchläuft verschiedene Schritte und wird als ein iterativer Prozess verstanden [7, 27, 32]. Anhand folgender Aktionen können komplexe Interventionen entwickelt werden [27]:

- Vorbereitung und Planung des Prozesses,
- Auswahl der Interessenvertreter:innen, die an der Entwicklung beteiligt werden sollten (Kliniker:innen, Kostenträger, Betroffene etc.),
- Gründung eines Entwicklungsteams,
- Überblick über die bestehende Evidenzlage (idealerweise anhand systematischer Übersichten),
- Berücksichtigung bereits bestehender Theorien zu Mechanismen oder Interventionswirkungen,
- Entwicklung eines Wirkmodells (*„program theory“*),
- erste Datenerhebung (Berücksichtigung qualitativer und quantitativer Methoden zur Kontextbestimmung bzw. zu vorläufigen Ergebnisparametern),
- Entwicklung eines Verständnisses zum Kontext, in den die zukünftige komplexe Intervention implementiert werden soll,
- Berücksichtigung des Kontexts zur späteren Implementierung (Möglichkeiten und Herausforderungen des zukünftigen Einsatzfelds) *und*
- Erarbeitung und Anpassungen des Interventionsansatzes (als iterativer Ansatz ohne klare Festlegung, ab wann die Intervention als vollständig angesehen werden kann).

Im Anschluss daran erfolgen Überprüfungen zur Anwendbarkeit (*„feasibility“*, gern auch in Form von Pilotierungen) sowie folgend eine prozessorientierte Evaluation mit weitergehendem Fokus auf der Überprüfung der neuen Intervention bezüglich Effizienz, Effektivität und Implementierungsreinheit sowie Wirkungen durch bzw. auf Kontextfaktoren [25, 32]. Diesbezüglich wird die Bedeutung des bereits in der Entwicklungsphase zu berücksichtigenden vorformulierten Wirkmodells, der Rahmenbedingungen für die spätere Anwendung sowie erforderlicher Ergebnisparameter zur Überprüfung als essenziell hervorgehoben [27, 32].

### Rahmenbedingungen für die neuen Interventionen E‑IMST und B‑IMST

Primäre Zielstellung des Entwicklungsprozesses ist es, ausgehend von der bisherigen Evidenzlage ein interdisziplinäres multimodales Therapieprogramm zu entwickeln, das niederschwellig und dadurch vor allem im ambulanten Versorgungssektor angeboten werden kann. Zur Evidenz interdisziplinärer, präventiver Ansätze sowie zu einer detaillierten Beschreibung ambulanter interdisziplinärer Versorgung in Deutschland wird auf die Arbeit von Kaiser et al. (2021; [15]) verwiesen.

Die IASP definiert *interdisziplinäre* Behandlungskonzepte als *multimodale* Therapieformen, die von einem *multidisziplinären* Behandlungsteam *integrativ* umgesetzt werden und ein *gemeinsames* biopsychosoziales Modell sowie daraus abgeleitete Ziele verfolgen [13]. Für Deutschland liegen weitergehende Konzeptionen vor [1, 2]. Sie verstehen unter interdisziplinärer multimodaler Schmerztherapie „die gleichzeitige, inhaltlich, zeitlich und in der Vorgehensweise aufeinander abgestimmte umfassende Behandlung von Patient:innen mit chronifizierten Schmerzsyndromen […], in die verschiedene somatische, körperlich übende, psychologisch übende und psychotherapeutische Verfahren nach vorgegebenem Behandlungsplan mit identischem, unter den Therapeut:innen abgesprochenem Therapieziel eingebunden sind“ [2]. Der integrative Behandlungsansatz vereint ein Behandlungsteam aus Ärzt:innen unterschiedlicher Fachrichtungen, Psycholog:innen bzw. Psychotherapeut:innen und Vertreter:innen zusätzlicher Disziplinen wie Physio‑, Ergo- oder Mototherapie, wobei evidenzbasierte Anwendungen integriert werden. Alle beteiligten Therapieformen stehen gleichberechtigt nebeneinander. In regelmäßigen Teambesprechungen werden Behandlungsziele gemeinsam festgelegt, angepasst und verfolgt. Insgesamt wird ein ressourcenorientiertes Vorgehen vorgeschlagen [2].

Die Entwicklung komplexer Interventionen wird als iterativer Prozess verstanden

Der Kontext der Anwendung von NVF wird demzufolge bereits durch bestehende Konzeptionen mitbestimmt [1, 2, 28]. Diese sind primär für die Diagnostik und Behandlung chronischer Schmerzverläufe entstanden. Dennoch sollen sie für die Übernahme in die Behandlung von Betroffenen mit wiederkehrenden Schmerzen und Risikofaktoren einer Chronifizierung berücksichtigt und geprüft werden.

Die interdisziplinäre Versorgungsform stellt die Umsetzung vor hohe prozessuale und strukturelle Herausforderungen. Eine frühzeitige Berücksichtigung des Kontexts einer späteren Implementierung, wie in den Empfehlungen vorgesehen, erscheint hier besonders wichtig.

Der zeitliche Rahmen beider zu konzipierender Gruppenformen orientierte sich an pragmatischen Überlegungen, in die sowohl klinische Erfahrung als auch Erwägungen zu den möglicherweise zumutbaren Bedingungen einflossen. Für die edukative Form (E‑IMST) wurde ein einmaliger Termin von 3 h angedacht, für die begleitende Form (B‑IMST) 10 Termine à 3 h zzgl. Einzelsitzungen über 10 Wochen. Die B‑IMST wurde als Intervention in geschlossener Gruppe geplant, um Gruppenkohäsion, Entwicklungspotenzial durch Konstanz in der Bearbeitung von persönlichen Inhalten sowie Planbarkeit der Inhalte über die 10 Wochen zu gewährleisten (Tab. 1). Die Entscheidung für die Durchführung der B‑IMST in einer geschlossenen Gruppe wurde intensiv im Expert:innenteam diskutiert und konsentiert.

**Table 1:** 

	Offenes Gruppenformat	Geschlossenes Gruppenformat
Wissensvermittlung	Durch Fluktuation der Mitglieder in der Planbarkeit beeinträchtigt; stufenweise Informationsvermittlung entsprechend dem Wissensstand der Teilnehmenden nicht gut möglich	Sukzessiver Aufbau von Wissen möglich; damit Veränderung von Sichtweisen und Haltungen der Teilnehmenden eher wahrscheinlich
Flexibilität in der Planung von Gruppensitzungen/Gruppen	*Höher*	*Geringer*
	Ausfälle leichter kompensierbar	Ausfälle nicht leicht kompensierbar
	Gruppenmitglieder schneller aufzunehmen	Gruppenmitglieder nur zu Beginn aufzunehmen, d. h. längere Wartezeiten
	Bessere Wirtschaftlichkeit	Geringere Wirtschaftlichkeit bei länger laufenden Gruppentherapien
Individuelle Entwicklungsmöglichkeit für die Gruppenmitglieder	*Geringer* Teilnahme noch Unbekannter hebt die Schwelle der Bereitschaft, sich mitzuteilen	*Höher* Durch intensivere Begleitung und Austausch von sich zunehmend bekannten Personen (Behandelnde/Behandelte)
Therapeutische Verbindlichkeit in der Vereinbarung zur Umsetzung von Transfer- und weiterführenden Aufgaben	*Geringer* Kein fester Rhythmus in der Teilnahme	*Höher* Fester Rhythmus in der Teilnahme sowie zunehmende Vertrautheit und Bekanntheit auf behandelnder und behandelter Seite

### Zielgruppen der neuen Versorgungsformen E‑IMST und B‑IMST

Entsprechend den Empfehlungen der Nationalen VersorgungsLeitlinie (NVL) „Nicht-spezifischer Kreuzschmerz“ [3] sollen sich bereits interdisziplinäre Versorgungsformen auch an Betroffene richten, die noch keine chronischen Schmerzen entwickelt haben.

Zielpopulation für das IMA [15]:

- Erwachsene (Alter ab 18 Jahre),
- Schmerzen seit mehr als 6 Wochen oder Schmerzrezidive trotz fachspezifischer Behandlung,
- Einschränkung des Lebensvollzugs und der gesundheitsbezogenen Lebensqualität durch den Schmerz,
- mit Risiko zur Chronifizierung der Schmerzen (Tab. 2) *und*
- ggf. aktuelle Arbeitsunfähigkeit seit 4 Wochen bzw. kumulierte Arbeitsunfähigkeit von mindestens 6 Wochen in den vergangenen 12 Monaten.

**Table 2:** 

**Somatische Risikofaktoren**	Aktuelle Arbeitsunfähigkeit seit 4 Wochen bzw. kumulierte Arbeitsunfähigkeit von mindesten 6 Wochen in den vergangenen 12 Monaten Bzgl. Lokalisation sich ausbreitende Schmerzen Hinweise auf Somatisierung (z. B. vielfältige, „bunte“ Symptomatik)
**Psychologische kognitiv-behaviorale Risikofaktoren**	*Ausgeprägtes (verbales/nonverbales) Schmerzverhalten* *Schmerzfördernde Schmerzverarbeitung (Fokussierung, Ängste …)* *Schmerzfördernde, krankheitsaufrechterhaltende Verhaltensweisen:* Ausgeprägtes Schon- und Vermeidungsverhalten Überforderung, „Durchhalten“ Hohes Inanspruchnahmeverhalten im Versorgungssystem, Wunsch nach fortgesetzter Krankschreibung bzw. fortgesetzter Diagnostik
**Psychologisch-affektive Risikofaktoren**	Depressive Symptome im Erleben und/oder Verhalten Befindlichkeit geprägt durch Frustration/Ärger
**Soziale Risikofaktoren**	Hinweise auf Stressbelastung in Familie/Partnerschaft/sozialem Umfeld/Beruf

Ausgehend davon wurden zwei Szenarien der vorchronischen Charakteristika möglicher Zielgruppen für gruppenbasierte Angebote angenommen:

#### Zielgruppe 1.

- Gering- bis mittelgradige Chronifizierung (Mainz Pain Staging System [MPSS] I–II) *und*
- gering- bis mittelgradige Beeinträchtigung durch bestehende, wiederkehrende Schmerzen (von Korff bis 2) *und*
- geringe Komplexität der Störung (Einfluss‑/Risikofaktoren bestehen auf den Ebenen des biopsychosozialen Modells zur Schmerzentstehung und sind entsprechend der Anamnese noch nicht manifestiert)

Für diese Zielgruppe kann davon ausgegangen werden, dass eine einmalige Edukation ausreichend ist.

#### Zielgruppe 2.

Auch hier ist davon auszugehen, dass noch kein manifestes Chronifizierungsgeschehen besteht, allerdings der Chronifizierungsprozess bereits weiter fortgeschritten ist. Dieses Szenario wird charakterisiert durch

- gering- bis mittelgradige Chronifizierung (MPSS I–II) *und*
- bereits höhere Beeinträchtigung durch bestehende, wiederkehrende Schmerzen (von Korff bis 4) *und*
- eine höhere Komplexität der Störung (ausgeprägte Einfluss‑/Risikofaktoren auf den Ebenen des biopsychosozialen Schmerzmodells, ohne dass die Patient:innen nach interdisziplinärer Einschätzung eine höher-intensive [teil-]stationäre IMST benötigen).

Eine strikte Abgrenzung innerhalb dieser letzten Gruppe gegenüber einer bereits manifesten chronischen Schmerzerkrankung, die entsprechend den Empfehlungen der Deutschen Schmerzgesellschaft e. V. durch intensive interdisziplinäre multimodale Ansätze versorgt werden soll, ist nur schwer möglich, weil ein Chronifizierungsgeschehen als fortschreitender, multifaktorieller Prozess anzusehen ist. Aus diesem Grund erfolgt die Indikationsstellung für ein ambulantes, interdisziplinäres präventives Therapieprogramm ausschließlich auf Basis eines qualifizierten interdisziplinären Assessments [5, 15, 28], ggf. im Rahmen des ambulanten IMA (A-IMA; https://www.a-ima.de) oder im Rahmen anderer etablierter Formen der Versorgung.

### Wirkmodell („program theory“)

Um die Wirkung einer Intervention überprüfen zu können, bedarf es der Formulierung eines Wirkmodells, das sich – in Abhängigkeit von theoretischen Annahmen und Vorüberlegungen – aus einem Veränderungsmodell (kausale Wirkprozesse) und einem Aktionsmodell (Bedingungen und Aktivitäten zur Wirkung einer Maßnahme) ableitet. Im Wirkmodell werden sowohl die angestrebten maßnahmenspezifischen als auch die abzusehenden Neben- und Folgewirkungen erfasst. Ergänzend dazu enthält das Wirkmodell diejenigen Prozesse, auf deren Basis die Wirksamkeit der Intervention angenommen wird. Abschließend kann im Rahmen eines Wirkmodells modelliert werden, in welcher Form ein Transfer der vermittelten Fähigkeiten und Fertigkeiten erwartet werden kann [39].

Ausschlaggebend für die zu konzipierende NVF sind die Risikofaktoren, die bisher in der Literatur bekannt sind [3] und mit der Chronifizierung von Schmerzen in Verbindung gebracht werden. Diese sind in Tab. 2 aufgeführt.

Präventive Strategien für die Vermeidung von Schmerzchronifizierung sind noch wenig untersucht. Eine Mischung aus Edukation und körperlich aktivierenden Übungen gilt vor allem in Bezug auf Schmerzreduktion und Reduktion von Beeinträchtigung bei Rückenschmerzen als effektiv [3]. Es ist jedoch davon auszugehen, dass diese Interventionen auch bei anderen wiederkehrenden Schmerzen nützlich sind.

Für Gruppentherapieinterventionen gibt es darüber hinaus Wirkfaktoren, die als unspezifisch benannt sind [10], sie sind in Tab. 3 zusammengefasst. Unter Wirkfaktoren werden dabei Prozesse verstanden, die in der Interaktion zwischen Patient:in und Therapierenden stattfinden. Sie stellen ein Zusammenspiel aus dem Verhalten der Therapierenden, die den Wirkfaktor aktivieren, und den Prozessen, die dadurch bei den Patient:innen angeregt werden, dar [10]. Diese unspezifischen Wirkfaktoren sollen zusätzlich in der Konzeption der gruppenbasierten NVF berücksichtigt werden.

**Table 3:** 

Wirkfaktor	Erläuterung
Problembewältigung	Die Hilfe zur Problembewältigung ermöglicht eine positive Bewältigungserfahrung im Umgang mit den Problemen der Patienten und Patientinnen (im Sinne eines verbesserten Selbstwirksamkeitserlebens)
Motivationale Klärung	Ermöglicht den Patienten und Patientinnen, sich selbst zu verstehen und sich über Werte, Ziele und Motive klarer zu werden und die bewussten und unbewussten Ziele, Erwartungen und Werte zu erforschen, die dem eigenen Erleben, Empfinden und Verhalten zugrunde liegen
Problemaktualisierung	Ermöglicht über die reale Erfahrung das Erlebbarmachen von Problemen, die der Patient/die Patientin in die Therapie mitbringt. Dies führt zu einer erhöhten Veränderungsbereitschaft
Ressourcenaktivierung	Beinhaltet die Stärkung und Förderung von Ressourcen bei den Patienten und Patientinnen, z. B. von Motivation, Fähigkeiten und Interessen
Therapeutische Beziehung	Meint die Qualität der Beziehung zwischen Patient:in und Therapierenden, die maßgeblich zum Erfolg einer Therapie beiträgt, indem die Therapierenden emphatisch, akzeptierend und wertschätzend den Patient:innen gegenübertreten und in ihnen positive Selbstschemata stärken. Das fördert u. a. auch das Erreichen einer positiven Therapieerwartung und Adhärenz

Ein spezifisches, auf die begleitende, intensivere Form der Gruppentherapie hin konzipiertes Wirkmodell wurde auf Grundlage der Ergebnisse aus den Workshops sowie der aktuell verfügbaren Literatur erarbeitet (siehe Ergebnisse, Abb. 6).

### Einbindung von Interessenvertretern und -vertreterinnen

#### Konzeptionsteam.

Die Koordinierung und Erarbeitung der Konzeption sowie des gesamten Manuals wurde im Rahmen des Projekts PAIN2020 [15] von einem multiprofessionellen Team der Konsortialpartner (Dresden, Mainz, Göttingen) vorgenommen. Dieses Team umfasste die Berufsgruppen Medizin, Physiotherapie und Psychotherapie. Die Aufgaben des Konzeptionsteams umfassten

- die Vorbereitung von Arbeitsmaterial für die Diskussion und Entscheidung durch die konsultierten Interessenvertreter:innen,
- die Aufbereitung der Ergebnisse aus diesen Diskussionen sowie
- die Überführung der Ergebnisse in ein Handbuch einschließlich Arbeitsmaterialien und Behandlerablaufpläne zur Durchführung der NVF.

Diese wurden erneut den konsultierten Interessenvertreter:innen vorgelegt, bis ein in dieser Gruppe akzeptierter Stand erreicht war.

#### Konsultierte Interessenvertreter:innen.

Je nach Phase wurden verschiedene weitere Interessenvertreter:innen aus Klinik und Wissenschaft, vornehmlich aus dem Kreis der Deutschen Schmerzgesellschaft e. V. sowie aus physiotherapeutischen und psychologischen Fachgesellschaften kontaktiert und in den Konzeptionsprozess eingebunden. Dabei wurde darauf geachtet, dass die professionsspezifischen Inhalte (Medizin, Physiotherapie, Psychologie) jeweils mit den Vertreter:innen der Fachgesellschaften erarbeitet und durch sie freigegeben wurden. Teamübergreifende Inhalte wurden aus dem PAIN2020-Team heraus erarbeitet und freigegeben.

### Ablauf der Konzeptionserstellung

**Figure 2:**
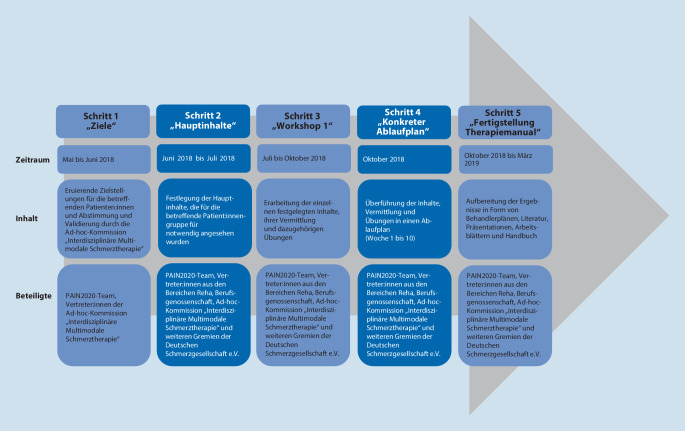

Aus Abb. 2 ergibt sich ein Überblick über den Gesamtprozess zur Erstellung der Konzeption sowie des Handbuchs einschließlich Manuale und Arbeitsmaterialien für die neue Versorgungsleistung. Dabei lag der zeitliche Schwerpunkt darauf, die begleitende Form einer interdisziplinären Gruppentherapie für die Zielgruppe 2 (stärker beeinträchtigte Personen) zu entwickeln, aus der heraus dann für Zielgruppe 1 Inhalte herausgelöst wurden.

Die Konzeption erfolgte in 5 Schritten (Abb. 2).

#### Schritt 1

Ausgehend von der Charakterisierung der Zielgruppen wurden zuerst Zielstellungen erarbeitet, die sich analog den Empfehlungen der NVL [3] ergaben und für PAIN2020 konkreter ausformuliert wurden. Diese Ziele wurden der Ad-hoc-Kommission „Interdisziplinäre Multimodale Schmerztherapie“ der Deutschen Schmerzgesellschaft e. V. zur Abstimmung und Diskussion in einem einmaligen Online-Survey vorgelegt. Die Kommission wurde gebeten, die Ziele zu gewichten, einerseits nach Wichtigkeit in Bezug auf die beiden Zielgruppen, andererseits hinsichtlich der Notwendigkeit, diese Ziele eher störungsspezifisch oder übergreifend (für alle üblichen Schmerzerkrankungen zusammenfassend) umzusetzen.

Die Befragung fand vom 02.05.2018 bis zum 07.05.2018 statt. Die Mitglieder der Ad-hoc-Kommission „Interdisziplinäre Multimodale Schmerztherapie“ wurden per E‑Mail angeschrieben und zu einer anonymen online-basierten Befragung (SurveyMonkey) eingeladen. Sechs Kolleg:innen der Kommission haben auf diese Befragung geantwortet (Mitgliederanzahl *n* = 17).

#### Schritt 2

Auf Grundlage der erarbeiteten Zielstellungen für die jeweiligen Zielgruppen wurden entsprechende Hauptinhalte zwischen Juni und Juli 2018 festgelegt. Die Koordination dieses Schritts oblag dem multidisziplinären Team aus PAIN2020, das vorbereitende Recherchen zu Mechanismen, bestehenden Manualen etc. durchführte, diese für die Diskussion aufbereitete und die Ergebnisse zusammenführte. Teilweise standen für die Erarbeitung von Inhalten bereits publizierte Konzepte, evidenzbasierte Modelle und Manuale zur Verfügung. Diese wurden in der grundlegenden Sichtung und Diskussion berücksichtigt.

In drei Videokonferenzen wurden Mitglieder der Ad-hoc-Kommission „Interdisziplinäre Multimodale Schmerztherapie“ und der Ad-hoc-Kommission „Curriculum Spezielle Schmerz-Physiotherapie“ (SpSPT) sowie von psychologischer Seite Mitglieder der Deutschen Gesellschaft für psychologische Schmerztherapie und -forschung e. V. (DGPSF) und für Rehabilitation und Gesundheitsförderung (DPA) um Feedback, Anregungen und Veränderungsvorschläge gebeten.

#### Schritt 3–5

Schritt 3–5 erfolgten aufeinander bezogen und in mehreren Runden iterativ, zuerst bei der intensiveren Gruppentherapieform für Zielgruppe 2 (stärker belastete Versicherte). Dabei wurde der bereits vorgesehene Rahmen von 10 Terminen à 3 h zzgl. Einzelterminen berücksichtigt.

Auftakt zu Schritt 3 bildete ein einmaliger Workshop in Präsenz zur *inhaltlichen und konzeptionellen Ausgestaltung der Therapieangebote* E‑IMST und B‑IMST. Dazu wurden die Ad-hoc-Kommission „Interdisziplinäre Multimodale Schmerztherapie“, ein multiprofessionelles Team der Konsortialpartner des Projekts PAIN2020 und weitere ärztliche, physiotherapeutische und psychologische Kolleg:innen aus Gremien der Deutschen Schmerzgesellschaft e. V. mit Erfahrungen in der IMST einbezogen.

Im Anschluss an den Workshop erfolgte die Konkretisierung und Präzisierung der Inhalte, ihrer Vermittlung und der dazugehörigen Übungen (Schritt 3, Juli–Oktober 2018).

Dazu wurden die zu konsultierenden Interessengruppen professionsspezifisch über Videokonferenzen mehrfach kontaktiert; teambezogene Inhalte wurden vom PAIN2020-Team besprochen. Aus diesen Inhalten entstanden sukzessive Ablaufschemata, die später in einem Handbuch zur Therapiedurchführung zusammengefasst wurden (Schritt 4, Ende Oktober 2018, als Ergebnis aus Schritt 3). Schlussendlich wurden aus den vorhergehenden Ergebnissen zwischen Oktober 2018 und März 2019 ein finales Manual zur Durchführung, Ergänzungen zur weiterführenden Literatur, Präsentationen für die Gruppendurchführung, Arbeitsblätter für die Patient:innen sowie Dokumentationsvorlagen der begleitenden ambulanten interdisziplinären multimodalen Gruppentherapie (B‑IMST) erarbeitet, die ebenfalls im Handbuch zur Therapiedurchführung zusammengefasst wurden.

Entsprechend den in Schritt 1 und 2 festgelegten Zielstellungen und Grundinhalten für die Zielgruppe 1 (bisher noch nicht schwer belastet durch die wiederkehrenden Schmerzen) wurden aus den vorliegenden Inhalten und Materialien während der Schritte 3–5 analog zur B‑IMST Manual, Material etc. für die E‑IMST erarbeitet sowie im Handbuch zusammengefasst. Das Handbuch kann auf Anfrage bei der Autorenschaft eingesehen werden.

Während der Schritte 3–5 entstanden auch ein Konzept für die Überprüfung der Umsetzbarkeit (*„feasibility“*), eine vorläufige Konzeption eines Wirkmodells sowie Parameter zur Beschreibung der Strukturen, Prozesse und Zufriedenheit sowohl der Patient:innen als auch der Behandler:innen, die im Rahmen der Pilotierung (Durchführung der Gruppentherapien im Rahmen von PAIN2020, Start April 2019) eingesetzt, durch ein intensives Monitoring begleitet und später ausgewertet wurden ([30], separate Publikation dazu in Vorbereitung).

## Ergebnisse

### Zusammenfassung der Ergebnisse aus der Befragung zu den Zielstellungen für E‑IMST und B‑IMST (Schritt 1)

**Figure 3:**
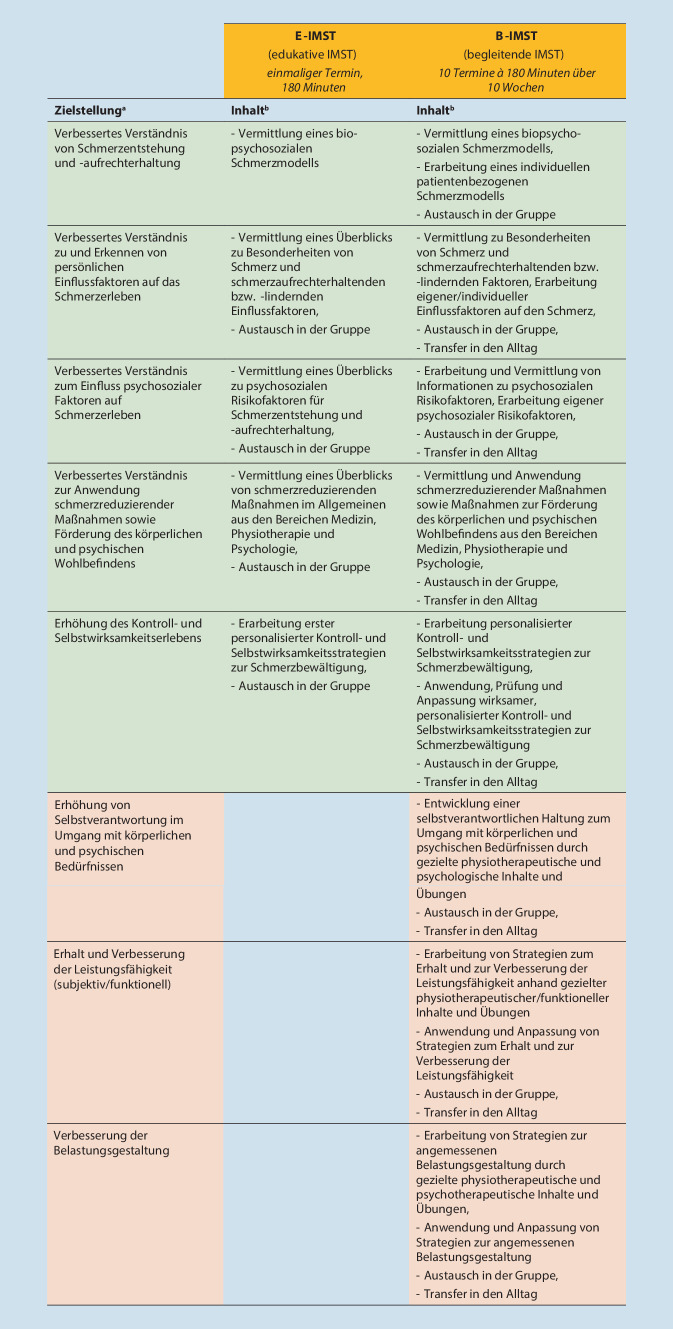

Schwerpunkte der Zielstellungen für die E‑IMST und B‑IMST sind in Abb. 3 dargestellt.

Grundlegende Übereinstimmung in der Zielstellung zwischen den beiden Therapieformen liegt in der

- Verbesserung des Verständnisses von Schmerz und mitbedingenden Faktoren,
- Erhöhung des Kontroll- und Selbstwirksamkeitserlebens bzw.
- Erhöhung von Eigenverantwortung in der Anwendung schmerzreduzierender Strategien.

Diese Ziele gehen auf die grundlegenden Ziele interdisziplinärer Schmerztherapie zurück [1, 2]. Unterschiede zwischen den beiden Therapiemodulen ergeben sich hinsichtlich weiterführender Ziele, die entsprechend den Bedarfen der Zielgruppen sowie in Abhängigkeit von den Rahmenbedingungen (einmalig vs. mehrmalig) variieren.

Überlegungen aus der Befragung der Ad-hoc-Kommission „Interdisziplinäre Multimodale Schmerztherapie“ in Bezug auf die *E‑IMST* zeigten auf, dass sowohl Schmerzlinderung als auch die funktionale Wiederherstellung als innerhalb eines einmaligen Informationstermins nicht umsetzbar verstanden werden. Es wurde stattdessen auf die neben der E‑IMST mögliche spezifische und begleitende Behandlung im jeweiligen Fachbereich (z. B. Physiotherapie) verwiesen. Die Ziele wurden, bezogen auf die Reichweite/Möglichkeiten der E‑IMST, als Vermittlung von Wissen und Anregungen definiert.

Ergänzend zu den bereits aufgeführten Zielen (Abb. 3) wurde als Zielstellung die Verbesserung bzw. Aufrechterhaltung der Funktionsfähigkeit, u. a. auch die Wiederaufnahme bzw. Beibehaltung der beruflichen Tätigkeit, als Schwerpunkt der B‑IMST definiert.

### Zusammenfassung der Ergebnisse aus der Erarbeitung relevanter Inhalte zur Umsetzung der Zielstellung für beide Zielgruppen (Schritt 2)

Die in Schritt 1 definierten Zielstellungen wurden als Grundlage für die inhaltliche Ausgestaltung herangezogen. Einerseits wurden Vorüberlegungen aus der klinischen Erfahrung heraus getroffen, andererseits wurde anhand festgelegter Stichworte (u. a. IMST, Schmerz, ambulante Therapieformen, Prävention, Therapieprogramme Schmerzbehandlung) nach fundierter und evidenzbasierter Literatur recherchiert. Allerdings zeigte sich diese Recherche als wenig ergiebig (siehe Einleitung) hinsichtlich bestehender Evidenz zu Interventionen in der hier anvisierten Zielgruppe. Daher wurde vor allem Wert auf bestehende, konsensbasierte Empfehlungen (NVL) gelegt, um Zielstellungen und darauf aufbauende Interventionen zu entwickeln. Diese ersten Rechercheergebnisse wurden dem multiprofessionellen Team vorgelegt und gemeinsam diskutiert. Im Austausch mit dem Team konnten wesentliche Interventionsmodelle und bestehende Manuale herangezogen werden (Infobox 1).

#### Infobox 1 Verwendete Modelle, Materialien und Manuale für die Konzeption der B‑IMST und der E‑IMST hinsichtlich der Inhalte, Abläufe und Materialien

- Von Wachter H, Hendrischke A (2016) Psychoedukation bei chronischen Schmerzen: Manual und Materialien. Springer-Verlag, Berlin, Heidelberg [35]
- Mohr B, Korsch S, Roch S, Hampel P (2017) Störungsbild unspezifischer chronischer Rückenschmerz. In: Debora – Trainingsmanual Rückenschmerzkompetenz und Depressionsprävention. Springer-Verlag, Berlin, Heidelberg [24]
- Chenot J, Pfingsten M, Marnitz U, Pfeifer K, Kohlmann T, Lindena G, Schmidt CO (2019) Effekte einer risikoadaptierten Kurzintervention zur Prävention der Chronifizierung bei akuten Rückenschmerzen. Schmerz:33:226–235 [6]
- Küch D, Fibich L, Herbold D, Franke GH (2017) Manualisierung von DOLORES – psychologisches Programm zur schmerzbezogenen Resilienz. In: Arbeitskreis Klinische Psychologie in der Rehabilitation (BDP) (Hrsg.) Psychologische Interventionen bei Verlusten und Verletzungen. Deutscher Psychologen Verlag, Bonn [16]

Wir danken allen Autoren und Autorinnen für die Verfügbarmachung Ihrer Arbeiten und die großzügige Genehmigung, Anteile und Arbeitsblätter für die Gruppenkonzeption in PAIN2020 zu verwenden.

#### Infobox 2 PAIN2020-Team

- Thomas Isenberg, Dr. Gabriele Lindena, Katharina Augustin, Carolin Martin, Anja Waidner, Andre Möller (Deutsche Schmerzgesellschaft e. V., Berlin)
- Dr. Ursula Marschall, Catharina Schuhmacher (BARMER, Wuppertal)
- Prof. Dr. Thomas Kohlmann, Daniel Szczotkowski (Universitätsmedizin Greifswald, Abt. Methoden der Community Medicine, Institut für Community Medicine, Greifswald)
- PD Dr. Ulrike Kaiser, Dr. Anne Gärtner, Dr. Anke Preißler, Greta Hoffmann (B.Sc. PT), Julia Pritzke-Michael (Universitätsklinikum Carl Gustav Carus, Medizinische Fakultät an der Technischen Universität Dresden, UniversitätsSchmerzCentrum, Dresden)
- Prof. Dr. Frank Petzke, Prof. Dr. Michael Pfingsten, Leonie Schouten (M.Sc. PT), Karin Deppe (M.A.) (Universitätsmedizin Göttingen, Schmerzmedizin, Klinik für Anästhesiologie, Göttingen)
- PD Dr. Ulrike Kaiser (Klinik für Anästhesiologie und Intensivmedizin, Universitätsklinikum Schleswig-Holstein, Lübeck)
- Dr. Bernd Nagel, Beatrice Metz-Oster (M.Sc.-Psych.), Katja Schwenk (M.Sc.-Psych.), Lena Milch (Dipl.-Psych.), Jana Rensland (M.Sc.-Psych.) (DRK Schmerz-Zentrum Mainz, Mainz)

In Abb. 3 findet sich die finale Festlegung der Hauptinhalte zur Umsetzung der Zielstellung für die B‑IMST sowie für die E‑IMST. In der inhaltlichen Ausgestaltung unterscheiden sich die beiden Therapiemodule vor allem in Bezug auf die Möglichkeit der praktischen Anwendung und Anpassung von Bewältigungsstrategien und Informationen an die persönliche Situation der Teilnehmenden (Abb. 3).

### Zusammenfassung der Ergebnisse aus der Ausarbeitung von Ablauf, Manual, Material und Handbuch (Schritt 3–5)

Ausgehend von den Zielstellungen aus Schritt 1, den Hauptinhalten aus Schritt 2 und den klinischen Erfahrungen der Teilnehmenden am Workshop sowie in Anlehnung an die oben aufgeführten Manuale wurden die einzelnen Therapiesitzungen der B‑IMST nacheinander hinsichtlich ihrer Inhalte und Abläufe sowie der Arbeits- und Übungsmaterialien diskutiert.

Aus den Ergebnissen zur Konzeption der B‑IMST entstanden Abläufe, Manual und Material für die E‑IMST unter Berücksichtigung der gegebenen Rahmenbedingungen von einmalig 3 h.

Da sich dieser Prozess iterativ bewegte und die einzelnen geplanten Schritte in der Durchführung ineinandergriffen, werden sie hier übergeordnet nach den Ergebnissen (inhaltliche Ausgestaltung, Erarbeitung Ablaufschema und Material einschließlich Handbuch) zur besseren Nachvollziehbarkeit dargestellt.

#### Inhaltliche Ausgestaltung der B‑IMST

##### Rahmenbedingungen zur Durchführung.

Die B‑IMST wird als wöchentliche Gruppentherapie von jeweils 3 h über 10 Wochen zzgl. professionsspezifischer Einzelsitzungen geplant.

In der Umsetzung der B‑IMST steht die interdisziplinäre Zusammenarbeit im Vordergrund, die in PAIN2020 über die bisher übliche Form teilweise hinausgeht. Eine Teamsitzung ist jeweils zweiwöchentlich geplant und wird von allen Behandelnden gestaltet (Anwesenheitspflicht für die Therapierenden in der Gruppe). Darüber hinaus wird die interdisziplinäre Zusammenarbeit in der B‑IMST durch eine gemeinsame Gruppengestaltung umgesetzt, in der zumindest (fast) immer physiotherapeutische und psychologische Kolleg:innen vertreten sind (je 24 von 30 h), in 8 von 30 h zusammen mit den ärztlichen Kolleg:innen. Gruppeninhalte werden gemeinsam vermittelt, Fragen können sowohl spezifisch als auch übergreifend beantwortet werden. Das Abschlussgespräch wird von den ärztlichen Berufsgruppen gemeinsam mit den Patient:innen in einem persönlichen Gespräch gehalten (30 min).

Die genuin *ärztlichen Aufgaben* bestehen in der Verantwortung für die Behandlung, in der Beratung, Führung der Patient:innen (einschließlich Befundeinordnung, Verantwortung bei Symptomveränderung/-verschlimmerung) sowie der Edukation schwerpunktmäßig zu den Themen Schmerzentstehung, medikamentöse Schmerztherapie, Bewegungsapparat und Schlaf. Ärztliche Aufgabe ist zudem die Koordination der Abschlussbrieferstellung sowie die Kommunikation mit den Nachbehandelnden.

Die genuin *physiotherapeutischen Aufgaben* liegen in der Edukation schwerpunktmäßig zu folgenden Themen:

- Wechselwirkung zwischen Stress und körperlicher Aktivität
- Motorische Grundfähigkeiten
- Allgemeine Trainingsprinzipien
- „Graded activity“
- Ansätze für einen erholsamen Schlaf
- Ergonomie

Die praktischen Übungen und Anleitungen fördern die Körperwahrnehmung und die motorischen Grundfähigkeiten, kurze Bewegungs- und Aktivierungspausen unterstützen die Belastungsregulation. Ressourcenaktivierung erfolgt durch einen engen alltagsnahen Bezug sowie das Erleben von Freude an der Bewegung.

Genuin *psychotherapeutische Aufgaben* bestehen in der Edukation insbesondere zu folgenden Themen:

- Zusammenhang von Emotion, Kognition und Verhalten mit dem Schmerzerleben
- Schlafhygiene
- Resilienz und Achtsamkeit als protektive Faktoren

Die praktischen Inhalte fokussieren auf

- die Vermittlung von Bewältigungsstrategien zur Entspannung,
- alltagsnahe Achtsamkeitsübungen,
- die Förderung individueller Ressourcen sowie
- Emotions- und Bedürfnisregulationsstrategien.

Die jeweils professionsspezifischen Inhalte wurden im Expert:innengremium diskutiert, wobei sich die Inhalte auf bisher in der praktischen Erfahrung bewährte Methoden stützen. Allem voran soll eine Alltagsnähe und -relevanz hergestellt werden, die die Selbstfürsorge maßgeblich fördert.

Ein Schwerpunkt für die B‑IMST liegt jedoch vor allem in der integrativen Zusammenarbeit aller drei Berufsgruppen. Die Konzeption der (meist) gleichzeitigen Anwesenheit von ärztlichen, physiotherapeutischen und psychologischen Behandelnden in der Gruppentherapie unterstützt das Ineinandergreifen der Inhalte und Übungen und soll das biopsychosoziale Modell integrativ vorleben bzw. vermitteln. Relevante Wirkfaktoren im Sinne einer tragfähigen therapeutischen Beziehung, motivationalen Klärung sowie Problemlösung werden durch alle Berufsgruppen adressiert und entsprechend deren speziellen berufsspezifischen Hintergründen unterfüttert. Damit sollen die Patient:innen eine integrative Sicht auf ihr persönliches Schmerzerleben und langfristig tragfähige Strategien im Umgang mit dem Schmerz sowie zum Erhalt ihrer körperlichen und psychischen Funktionsfähigkeit erhalten.

##### Struktur und Ablauf.

**Figure 4:**
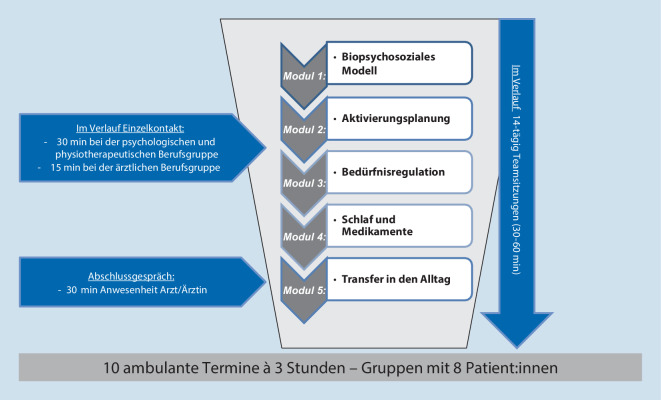

Die B‑IMST setzt sich aus 5 Modulen mit je 2 Terminen innerhalb von 2 Wochen zusammen. Die Therapiemodule unterliegen der in Abb. 4 dargestellten Reihenfolge und sind als Gruppenintervention angelegt. Als Grundlage für die Erarbeitung von Selbstmanagementfähigkeiten steht zu Beginn das Modul „biopsychosoziales Modell“. Den Abschluss bildet das Modul „Transfer in den Alltag“.

Die Differenzierung der Module in Themen wird in Tab. 4 dargestellt.

**Table 4:** 

***Biopsychosoziales Modell***		
**Modul 1**	*Woche 1*	Vorstellung der Therapierenden, des Konzepts und Ablaufs^a^ Biopsychosoziales Schmerzmodell^a^ Patientenspezifische Funktionsskala (PSFS)
	*Woche 2*	Anatomie und Physiologie^a^ Resilienz Graded-activity-Konzept
***Aktivierungsplanung***		
**Modul 2**	*Woche 3*	Stress und Schmerz^a^ Autonomes Nervensystem^a^
	*Woche 4*	Achtsamkeit^a^ Belastungssteuerung Ausdauer
***Bedürfnisregulation***		
**Modul 3**	*Woche 5*	Gedanken und Schmerz Segmentale Kontrolle Beweglichkeit
	*Woche 6*	Bedürfnisse, Emotionen und Schmerz Koordination und Kraft
***Schlaf und Medikamente***		
**Modul 4**	*Woche 7*	Schlaf und Schlafstörungen Bewegungssicherheit
	*Woche 8*	Medikamentöse Schmerztherapie Beratung zur körperlichen Aktivität^a^ Ergonomie
***Transfer in den Alltag***		
**Modul 5**	*Woche 9*	Rückfallprophylaxe Transferbezogene Zielformulierung
	*Woche 10*	Resümee und Verabschiedung der Patient:innen

*B‑IMST* begleitende interdisziplinäre multimodale Schmerztherapie, *E‑IMST* edukative interdisziplinäre multimodale Schmerztherapie

^a^Inhalte sind Bestandteile der E‑IMST

Jede Therapiesitzung vertieft zunächst theoretisch das jeweilige Thema, um es dann anhand konkreter Übungen und Hausaufgaben in die Erfahrungswelt der Patient:innen zu transferieren. Zudem beinhaltet jede Therapiesitzung eine Aktivierungs- und Sensibilisierungsphase, in der die Patient:innen angeregt werden, die Inhalte zu sich und ihrer Erfahrung in Beziehung zu setzen. Darauf baut der theoretische Inhalt auf, der idealerweise an den Erfahrungen der Patient:innen ansetzt. Jede Therapiesitzung endet mit praktischen Inhalten (Übungen, Diskussion, Hausaufgaben), die dann in der sich anschließenden Therapiesitzung in der folgenden Woche wieder aufgegriffen werden und in das neue Thema überleiten.

Die Themen (theoretische Grundlagen) werden durch edukative Vorträge anhand vorbereiteter Präsentationen eingeführt und durch praktische Übungseinheiten (Bewältigungsstrategien) vertieft. Für die Vorträge stehen Hintergrundinformationen, Literaturhinweise und umfangreiche Präsentationen sowie Arbeitsblätter und Übungsanleitungen zur Verfügung.

Über die Einbindung des Erlebens der Teilnehmenden durch Übungs- bzw. Erfahrungsanteile wird die Problemaktualisierung (siehe Wirkmodell) adressiert und die eigene Betroffenheit aktualisiert. Gleichzeitig entsteht ein Bezug zu den erlernten Strategien, die sich im Sinne einer Ressourcenaktivierung (siehe Wirkmodell) abbilden.

Im Rahmen *kurzer professionsspezifischer Einzelsitzungen* (1-mal 30 min für die physiotherapeutische und psychologische Berufsgruppe, 1‑mal 15 min im Verlauf für die ärztliche Berufsgruppe und zusätzliche 30 min Abschlussgespräch, Abb. 4) können neben berufsgruppenspezifisch vermittelten Informationen in der Gruppe persönliche Fragen gestellt und vonseiten der Berufsgruppen gezielte Maßnahmen mit den Teilnehmenden besprochen bzw. vertieft werden.

#### Inhaltliche Ausgestaltung der E‑IMST

##### Rahmenbedingungen.

Neben der Informationsvermittlung durch die jeweilige Profession in der Gruppe soll auch der übende Anteil einen Großteil der Zeit einnehmen. Hierbei befasst sich der erste Teil vor allem mit einer theoretischen Einführung, auf die hin im zweiten Teil mögliche Bewältigungsansätze vorgestellt werden.

Die E‑IMST ist ausschließlich als Gruppenintervention angelegt; berufsgruppenspezifische Einzelsitzungen sind nicht vorgesehen.

Die *ärztlichen Aufgaben* bestehen in erster Linie in der Vermittlung von Informationen zum biopsychosozialen Modell sowie zur Neurophysiologie des Schmerzes und zur allgemeinen Beratung bei Fragen. Die *physiotherapeutischen Aufgaben* liegen in Übungen zur Lockerung und Aktivierung. Als mögliche Bewältigungsstrategien werden die Themen Ausdauer, Dehnung und Mobilität informativ vorgestellt und beübt. *Psychotherapeutische Aufgabe* ist die Vorstellung von Bewältigungsansätzen, insbesondere durch das Thema Achtsamkeit, das mit einer kurzen Achtsamkeitsübung praktisch geübt wird. Integrativ durch *alle Berufsgruppen* wird gemeinsam mit den Patient:innen das Thema Stress und Stressreaktionen erarbeitet. Vor allem bezüglich körperlicher und psychischer Stressreaktionen werden Patienten und Patientinnen in die Diskussion einbezogen und der Austausch über persönliche Stresserfahrungen gefördert.

##### Struktur und Ablauf.

**Figure 5:**
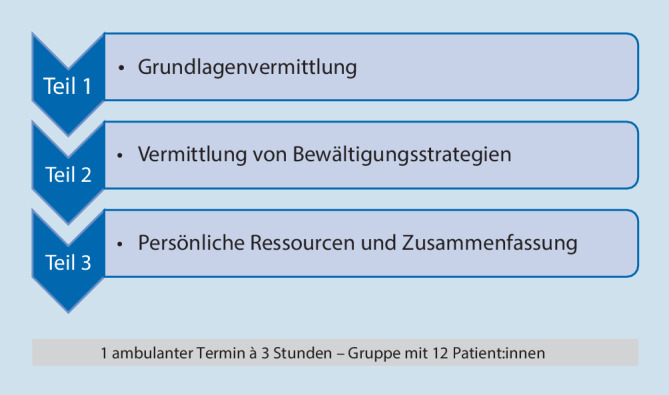

Die E‑IMST versteht sich in erster Linie als Intervention zur Vermittlung von Wissen über Schmerz und das biopsychosoziale Schmerzmodell (Abb. 5 bzw. Tab. 4). Um den Bezug zur eigenen Situation herstellen zu können, sollen die Erfahrungen der Patient:innen unmittelbar einfließen können. Aus diesem Grund kommen sowohl theoretische als auch praktische Interventionen bzw. erfahrungs- und erlebnisorientierte Methoden gleichermaßen zur Anwendung und adaptive Bewältigungsansätze sollen aus ärztlicher, physiotherapeutischer und psychologischer Sicht aufgebaut und erlernt werden.

Hinsichtlich der theoretischen und praktischen Inhalte bzw. der erfahrungs- und erlebnisorientierten Methoden wurden aus den Inhalten der B‑IMST wesentliche Kernelemente (u. a. biopsychosoziales Schmerzmodell, Stressmanagement, Medikamente) extrahiert und für die E‑IMST zusammengefügt. Neue, nur in der E‑IMST zur Anwendung kommende Inhalte wurden nicht erarbeitet.

#### Erarbeitung eines Manuals zur Gruppendurchführung

Für jedes Therapiemodul (E‑IMST und B‑IMST) bzw. für jede Therapiesitzung (B‑IMST) wurde ein Ablaufplan für Patient:innen sowie Behandelnde erarbeitet. Die Ablaufpläne der Behandelnden veranschaulichen neben den Themenbausteinen deren Reihenfolge und eine für diese Themen orientierende Zeiteinheit. Daneben werden die benötigten Arbeitsmaterialien vorgestellt sowie Hinweise zur Durchführung und zu weiterführender Literatur beschrieben. Ein Beispiel findet sich in Tab. 5.

**Table 5:** 

Woche	Modul	Ablaufplan/zu vermittelnde Inhalte	Dauer (min)	Übungsaufgaben für zu Hause	Benötigtes Arbeitsmaterial
1	*Modul 1:* Vermittlung eines biopsychosozialen Modells	1. Kennenlernen Therapeut:innen/Patient:innen, Gruppenregeln – *alle*	20	A3 Fischernetz A4 Aktivitätenprotokoll A5 PSFS für Patient:innen	*Präsentationen:* Wo1_Gesamtpräsentation *Arbeitsblätter:* A0 Gruppenregeln A1a Einflussfaktoren auf den Schmerz *oder* A1b Hemmer-Antreiber A2 Zitronenübung A3 Fischernetz A4 Aktivitätenprotokoll *Physiotherapie:* P1_Arbeitsmaterial Übungen_PT A5 PSFS für Patient:innen A6 PSFS für Gruppenstunde *Allgemein:* Karteikarten, Flipchart, Stifte Beamer/Laptop
		2. Konzeptvorstellung – *alle*	5		
		3. Erwartungen der Patient:innen – *alle*	15		
		Pause	5		
		4. Wahrnehmungsübung „Einflussfaktoren auf den Schmerz“ (Patient:innenbefragung) – *alle*	15		
		5. Thema biopsychosoziales Modell – *alle*	25		
		6. Thema Neurophysiologie – *Med*	30		
		7. Thema psychologische Aspekte der Schmerzwahrnehmung – *Psych*	15		
		Pause	10		
		8. Film: Zusammenfassung im biopsychosozialen Modell – *alle*	10		
		9. Einführung Thema PSFS – *PT*	10		
		10. Transferübungen: Arbeitsblatt „Fischernetz“, Aktivitätenprotokoll – *Psych*	10		
		11. Übung „Lockerungsübung“	10		
		Σ	180		

*PSFS* Patienten-spezifische Funktionsskala

Beteiligte Berufsgruppen: *Med* Medizin, *PT* Physiotherapie, *Psych* Psychotherapie

Für jede Sitzung werden Arbeitsblätter zur Bearbeitung durch die Patient:innen sowie PowerPoint-Präsentationen zur Informationsvermittlung und Gruppenstruktur zur Verfügung gestellt. Die an die Patient:innen ausgeteilten Arbeitsblätter werden als Angebote betrachtet und kommuniziert. Sie dienen sowohl der Erarbeitung von Inhalten in der Gruppenstunde als auch der Anleitung von Übungen und Vermittlung von Informationen, zudem sollen sie die Erinnerung und den Transfer in den Alltag unterstützen.

Die Festlegung von Arbeitsblättern und Präsentationen für die Durchführung der E‑IMST erfolgte durch Auswahl aus dem für die B‑IMST erstellten Material.

### Abschließende Aktivitäten und Vorbereitung der Pilotierungsphase

Zur Durchführung der Gruppentherapie E‑IMST/B‑IMST gibt es speziell für die zuständigen Behandelnden das *Handbuch „Gruppentherapie“*, um die Umsetzung in der jeweiligen Einrichtung zu erleichtern. In diesem werden kompakt und zusammenhängend alle nötigen Informationen zur Durchführung, zur Dokumentation und zum Monitoring in Bezug auf die Durchführung der Therapiemodule vorgestellt und zusammengefasst. Sowohl hinsichtlich Hintergrundliteratur für die eigene Vertiefung von Wissen zu den Modulinhalten als auch in Bezug auf physiotherapeutische Übungen enthält das Handbuch zusätzliches Material.

**Figure 6:**
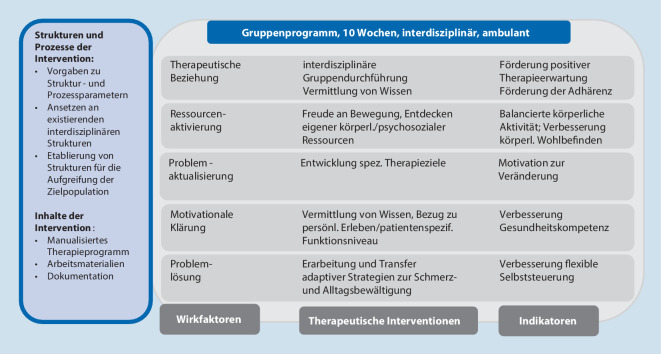

In Vorbereitung auf die Durchführung der Intervention im Rahmen von PAIN2020 wurde eine Pilotierung geplant, die diese neuen Interventionsformen prüft – vor allem in Bezug auf Umsetzbarkeit, Machbarkeit und Akzeptanz (sowohl durch Patient:innen als auch durch die Behandelnden). Dafür wurde ein *vorläufiges Wirkmodell* entwickelt, das in Abb. 6 dargestellt ist.

Dieses Wirkmodell führt

- die Ebenen allgemeiner Wirkfaktoren (beispielsweise therapeutische Beziehung, Ressourcenaktivierung),
- die speziell auf die Zielpopulation hin entwickelten (übergeordneten) therapeutischen Interventionen sowie
- die für die Prozessevaluation vorgesehenen Indikatoren

zusammen. Hinsichtlich der therapeutischen Interventionen wurden die aus der Literatur ersichtlichen Hinweise auf die förderliche Funktion von Edukation (motivationale Klärung: Vermittlung von Wissen, Bezug zu persönlichem Erleben und patientenspezifischem Funktionsniveau; therapeutische Beziehung: interdisziplinäre Gruppendurchführung zur Vermittlung von Wissen) und körperlicher Aktivierung (Ressourcenaktualisierung: Freude an Bewegung, Entdecken eigener körperlicher/psychosozialer Ressourcen) umgesetzt. Anhand der Indikatoren Verbesserung der Gesundheitskompetenz (Edukation), balancierte körperliche Aktivität sowie Verbesserung des körperlichen Wohlbefindens (körperliche Aktivität) sollen die eingesetzten Maßnahmen hinsichtlich ihrer Wirksamkeit überprüft werden.

Darüber hinaus wurden Kriterien für die Betrachtung der Durchführung der NVF E‑IMST und B‑IMST entwickelt, die für ein projektinternes Monitoring bzw. die Prüfung der Gruppeninterventionen unter Kontextbedingungen benötigt werden ([30]; Publikation zu diesen Ergebnissen in Vorbereitung).

## Diskussion

Entsprechend dem aktuellen Versorgungsstand und den Bedarfen der avisierten Zielpopulation (Patient:innen mit [häufig] wiederkehrenden Schmerzen und Risikofaktoren für eine Chronifizierung) wird die Erweiterung der Gesundheitsversorgung um frühzeitige, niederschwellige interdisziplinäre Versorgungsangebote gefordert [3, 8]. Primäre Zielstellung solcher Angebote ist die Vermeidung einer (weiter fortschreitenden) Chronifizierung von Schmerzen, die als Sekundärprävention bezeichnet werden kann. Damit sollen mittel- und langfristig Arbeitsunfähigkeit, Frühberentung und die Entwicklung komorbider psychischer und physischer Erkrankungen verhindert werden.

Mit Blick auf die Komplexität von Risiko- und Wirkfaktoren sowie deren Zusammenwirken sollten derartige Therapieansätze sowohl mechanismen- als auch grundlagenbasierte Theorien und Modelle vereinen und – naturgemäß – durch ein interdisziplinäres Team – im Sinne des IMST-Ansatzes – vermittelt werden. Die Schonung der zeitlichen, personellen und finanziellen Ressourcen der Versorger:innen sollte damit ebenso berücksichtigt werden wie die personellen und zeitlichen Aufwendungen aufseiten der Patient:innen. An diesem Punkt setzte das bundesweite Forschungsprojekt PAIN2020 (Patientenorientiert.Abgestuft.Interdisziplinär.Netzwerk, Förder-Nr. 01NVF17049) an. Im Rahmen der Sekundärprävention sollten Patient:innen frühzeitig im Krankheitsverlauf nach entsprechenden Kriterien eingeschlossen werden, um einer Chronifizierung der Schmerzen vorzubeugen.

Im Rahmen des Projekts PAIN2020 war es möglich, zwei entsprechende Interventionen konzeptuell zu entwickeln. Diese stehen nun als Handbuch mit allen erforderlichen Materialien zur Verfügung und können, so sich Umsetzbarkeit und Machbarkeit bzw. positive Wirksamkeitsnachweise unter Kontextbedingungen zeigen, in der Gesundheitsversorgung angewendet werden, beispielsweise im Rahmen des KBV-Vertrags (*KBV* Kassenärztliche Bundesvereinigung) oder im Rahmen des Disease-Management-Programms Rückenschmerz.

Der vorliegende Beitrag beschreibt ausschließlich die Entwicklung einer Konzeption für eine einmalige edukative Intervention (E‑IMST) sowie eine begleitende Intervention (B‑IMST), die beide interdisziplinär gedacht sind und die Qualitätskriterien für eine IMST erfüllen (u. a. Zusammenarbeit/Anwesenheit der Berufsgruppen oder Teamsitzungen). Damit ist der erste Schritt abgeschlossen, an den sich jedoch Machbarkeitsanalysen und in der Folge weitere Evaluationen anschließen müssen, die wiederum auch zu Änderungen an Konzeption und Manual führen werden.

Die Einbindung verschiedener erfahrener, multidisziplinärer Kliniker:innen sowie Wissenschaftler:innen war eine Herausforderung für den laufenden Prozess, führte aber aus Sicht der Autorenschaft zu einem gut fundierten Handbuch und Material.

Bei der Entwicklung beider Therapiemodule orientierte sich die vorliegende Arbeit durchgehend an den Schritten zur Konzeption einer komplexen Intervention (vgl. Methodik), was im Ergebnis des hier dargestellten Prozesses zur Formulierung eines vorläufigen Wirkmodells führte (*„program theory“*), das sich an den Wirkfaktoren von gruppen- und psychologisch basierten Interventionen orientierte und erste Strukturen und Prozesse sowie die Inhalte der therapeutischen Intervention der B‑IMST beschreibt. Hier bereits hinterlegte Indikatoren werden bei Machbarkeitsanalysen sowie Prozessevaluationen wichtig sein.

Das erarbeitete Wirkmodell kann aktuell als vorläufig betrachtet werden

Eine weitere Publikation zur Beschreibung der Ergebnisse der sich anschließenden Schritte ist in Erarbeitung, u. a. geht es hier um die Datenerhebung und Prüfung zur Anwendbarkeit (*„feasibility“*) innerhalb beider Therapiemodule und um die inhaltliche Erweiterung des Wirkmodells auf weitere Aspekte (u. a. weitere Strukturen und Prozesse, Outcome).

Erschwerend bei der Konzeption beider Interventionen war, dass aktuell noch wenige Studien für diese Zielgruppen und relevante Mechanismen existieren, noch weniger über wirksame Interventionen. Der Ansatz, sich über Risikofaktoren den Bedarfen dieser Zielgruppen zu nähern, ist ein erster Lösungsansatz – der entsprechend einer sich verändernden Evidenz angepasst und ergänzt werden sollte. Aufgrund der aktuell begrenzten Evidenz wurde daher auf Expert:innenerfahrung in der Schmerztherapie zurückgegriffen. Das Wirkmodell, das erarbeitet wurde, kann daher aktuell als vorläufig betrachtet werden, allerdings bietet es einen ersten Ansatz, die Wirkungen therapeutischer Interventionen in dieser Zielgruppe systematisch zu untersuchen und damit weitere Evidenz beizusteuern, die ggf. auch andere Schmerzformen wie Kopfschmerz berücksichtigt.

## Fazit für die Praxis

Es existieren nun zwei interdisziplinär ausgerichtete Manuale für Patient:innen mit Schmerzen unterschiedlicher Lokalisation, die u. a. eine Lücke in der Versorgung von Menschen mit wiederkehrenden Schmerzen und Risikoprofil für eine Chronifizierung füllen könnten und die bereits von Beginn an interdisziplinär ausgerichtet sind. Diese Ansätze müssen sich allerdings entsprechend den Empfehlungen für die Entwicklung komplexer Interventionen sowohl in der Machbarkeit als auch in ihrer Wirksamkeit bewähren. Eine Darstellung der Ergebnisse zur Machbarkeit der edukativen (E‑IMST) und begleitenden interdisziplinären multimodalen Schmerztherapie (B‑IMST) im Rahmen von PAIN2020 wird derzeit vorbereitet, wobei auch die Ergebnisse aus dem projektbezogenen Monitoring der Anwendung zwischen 2019 und 2022 einfließen und kritisch diskutiert werden. Darüber hinaus steht in dem neuen Projekt Patientenorientiert.Abgestuft.Interdisziplinär.Netzwerk – Therapie/PAIN2.0 (Förderung Innovationsfonds, Förder-Nr. 01NVF20023) die Überprüfung der Wirksamkeit der begleitenden interdisziplinären multimodalen Schmerztherapie (B‑IMST wird zur A‑IMST – ambulante interdisziplinäre multimodale Schmerztherapie) unter Kontextbedingungen im Vordergrund (Studienprotokoll in Vorbereitung, https://www.pain2punkt0.de).
